# A novel nested multiplex polymerase chain reaction (PCR) assay for differential detection of *Entamoeba histolytica*, *E. moshkovskii *and *E. dispar *DNA in stool samples

**DOI:** 10.1186/1471-2180-7-47

**Published:** 2007-05-24

**Authors:** Krishna Khairnar, Subhash C Parija

**Affiliations:** 1 Department of Microbiology, Jawaharlal Institute of Postgraduate Medical Education and Research (JIPMER), Puducherry, India

## Abstract

**Background:**

*E. histolytica*, a pathogenic amoeba, is indistinguishable in its cyst and trophozoite stages from those of non-pathogenic *E. moshkovskii *and *E. dispar *by light microscopy. We have developed a nested multiplex PCR targeting a 16S-like rRNA gene for differential detection of all the three morphologically similar forms of *E. histolytica*, *E. moshkovskii *and *E. dispar *simultaneously in stool samples.

**Results:**

The species specific product size for *E. histolytica*, *E. moshkovskii *and *E. dispar *was 439, 553 and 174 bp respectively, which was clearly different for all the three *Entamoeba *species. The nested multiplex PCR showed a sensitivity of 94% and specificity of 100% for the demonstration of *E. histolytica*, *E. moshkovskii *and *E. dispar *DNA in stool samples. The PCR was positive for *E. histolytica*, *E. moshkovskii *and *E. dispar *in a total of 190 out of 202 stool specimens (94% sensitive) that were positive for *E. histolytica/E. dispar*/*E. moshkovskii *by examination of stool by microscopy and/or culture. All the 35 negative control stool samples that were negative for *E. histolytica/E. dispar*/*E. moshkovskii *by microscopy and culture were also found negative by the nested multiplex PCR (100% specific). The result from the study shows that only 34.6% of the patient stool samples that were positive for *E. histolytica/E. dispar*/*E. moshkovskii *by examination of stool by microscopy and/or culture, were actually positive for pathogenic *E. histolytica *and the remaining majority of the stool samples were positive for non-pathogenic *E. dispar *or *E. moshkovskii *as demonstrated by the use of nested multiplex PCR.

**Conclusion:**

The present study reports a new nested multiplex PCR strategy for species specific detection and differentiation of *E. histolytica*, *E. dispar *and *E. moshkovskii *DNA in stool specimens. The test is highly specific, sensitive and also rapid, providing the results within 12 hours of receiving stool specimens.

## Background

Infection with *Entamoeba histolytica *results in 34 million to 50 million symptomatic cases of amoebiasis worldwide each year, causing 40 thousand to 100 thousand deaths annually [1]. *E. histolytica*, the pathogenic amoeba, is indistinguishable in its cyst and trophozoite stages from those of non-pathogenic *E. dispar *[1] and *E. moshkovskii *[2], except in rare cases of invasive disease when *E. histolytica *trophozoite may contain ingested red blood cells [3]. Estimates of intestinal *E. histolytica *infections have been primarily based on microscopic examination of stools, which has a sensitivity of only 60%, even under optimal standards [4,5]. The microscopic examination of stool alone, however, fails to differentiate *E. histolytica *from those of morphologically similar non-pathogenic species such as *E. dispar *and *E. moshkovskii*. Stool culture followed by isoenzyme analysis enables the differentiation of *E. histolytica *from *E. dispar *[6]. However, this method of culture when followed by isoenzyme analysis requires one to several weeks to obtain the result and also special laboratory facilities, making it impractical for use in the routine diagnosis of intestinal amoebiasis.

Various approaches are being followed for specific identification and detection of *E. histolytica *in stool specimens such as detection of *E. histolytica *coproantigen in stool samples by enzyme-linked immuno-sorbent assay (ELISA) and detection of *E. histolytica *DNA in stool samples by polymerase chain reaction (PCR). Currently few commercial ELISA kits are available for detection of *E. histolytica/E. dispar *coproantigen in stool. These include TechLab *Entamoeba *test to detect *E. histolytica/E. dispar *[5], Alexon ProSpecT ELISA to detect *E. histolytica/E. dispar *and *Giardia lamblia *[7] and Triage parasite panel to detect antigen of *E. histolytica/E. dispar*, *Giardia lamblia *and *Cryptosporidium parvum *in stool specimens [8]. The main limitation of all these ELISA kits is that they can identify the amoebae only as *E. histolytica*-*E. dispar *complex but not specifically as *E. histolytica*, *E. dispar *or *E. moshkovskii*. However, a monoclonal antibody based Tech Lab *E. histolytica *II ELISA is commercially available for the specific detection of *E. histolytica *antigen directly in stool specimen [9], but this kit can neither detect *E. dispar *nor *E. moshkovskii *in stool specimen.

Recently, a nested PCR targeting 16S-like rRNA gene has been reported from the International Centre for Diarrhoeal Disease Research, Dhaka, Bangladesh (ICDDR,B) [2] as well as from our laboratory at Jawaharlal Institute of Postgraduate Medical Education and Research (JIPMER) hospital, Puducherry, India [10] to detect and differentiate *E. histolytica*, *E. dispar *and *E. moshkovskii *directly in stool specimens. But the identification of the amoeba in the stool specimens either as *E. histolytica*, *E. dispar *or *E. moshkovskii *was carried out by performing nested PCR each time separately for individual species which was tedious. To overcome this disadvantage, the main objective of the present study was to develop and evaluate a nested multiplex PCR targeting the 16S- like rRNA gene for simultaneous detection and differentiation of *E. histolytica*, *E. moshkovskii *and *E. dispar *directly in stool samples.

## Results

### Microscopy and culture of stool

A total of 202 out of 1,720 stool samples screened were positive for *E. histolytica/E. dispar*/*E. moshkovskii *by microscopy and/or culture. These included 164 specimens positive for *E. histolytica/E. dispar*/*E. moshkovskii *by both microscopy and culture, 22 positive by microscopy and 16 positive by culture (Table 1). All the 35 negative control stool samples were negative for *E. histolytica/E. dispar*/*E. moshkovskii *by microscopy and culture.

### Nested multiplex PCR

#### Quantification of DNA in stool specimen

The DNA yield was found to be approximately 49 μg/ml. The purity of DNA extract from stool specimen was found to be satisfactory as the value of ratio of readings at 260 nm and 280 nm (OD _260_/OD _280_) was approximately 1.8.

#### Primer validation

The sequencing result of PCR product of *E. dispar*, *E. histolytica *and *E. moshkovskii *Laredo showed 99% to 100% identity to the sequences of *E. dispar*, *E. histolytica *and *E. moshkovskii *Laredo, deposited in GenBank with accession number [GenBank:Z49256], [GenBank:X56991] and [GenBank:AF149906] respectively.

#### Assessment of competition for non-target DNA

The assessment of competition of other non-target DNA present in stool DNA extract with a target DNA in nested multiplex PCR showed expected amplification without any non-specific amplification.

#### Estimation of minimum number of *Entamoeba *cells detectable by nested multiplex PCR

**Figure 1:**
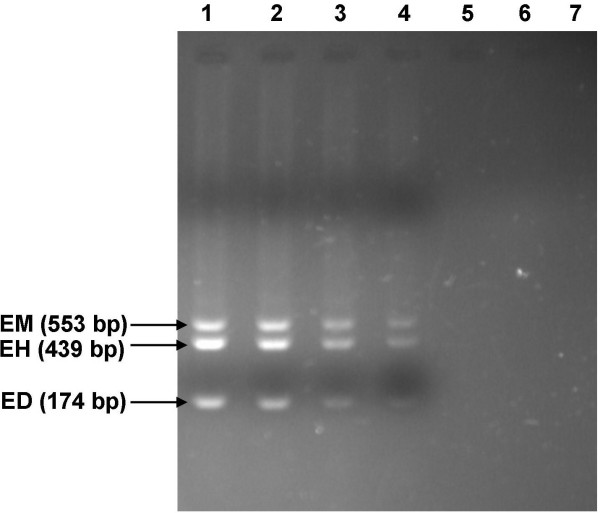
Sensitivity of PCR for detection of minimum number of *E. moshkovskii *(EM), *E. histolytica *(EH) and *E. dispar *(ED) cells. 0.05 gm of negative control stool specimen seeded with serially diluted *Entamoeba *cells corresponding to 10^6 ^cells (lane 1), 10^5 ^cells (lane 2), 10^4 ^cells (lane 3), 10^3 ^cells (lane 4), 10^2 ^cells (lane 5), and 10 cells (lane 6) were subjected to DNA extraction followed by PCR amplification. Amplified products were analyzed by agarose gel electrophoresis. The sizes of the amplification products are indicated on the left (in base pairs). Lanes 7, negative control (PCR without DNA)

The nested multiplex PCR detected *E. histolytica*, *E. dispar *and *E. moshkovskii *DNA, even at the minimum parasite concentration tested (1000 parasites/0.05 grams of faeces). The detection limit of nested multiplex PCR for *E. histolytica*, *E. dispar *and *E. moshkovskii *was found to be approximately 25 *Entamoeba *protozoa cells, since 2.5 μl of template DNA (1000 parasite/100 μl of TE buffer) gave positive signal (Figure 1).

#### Estimation of nested multiplex PCR to detect mixed infections with *E. histolytica*, *E. dispar *and *E. moshkovskii *species

**Figure 2:**
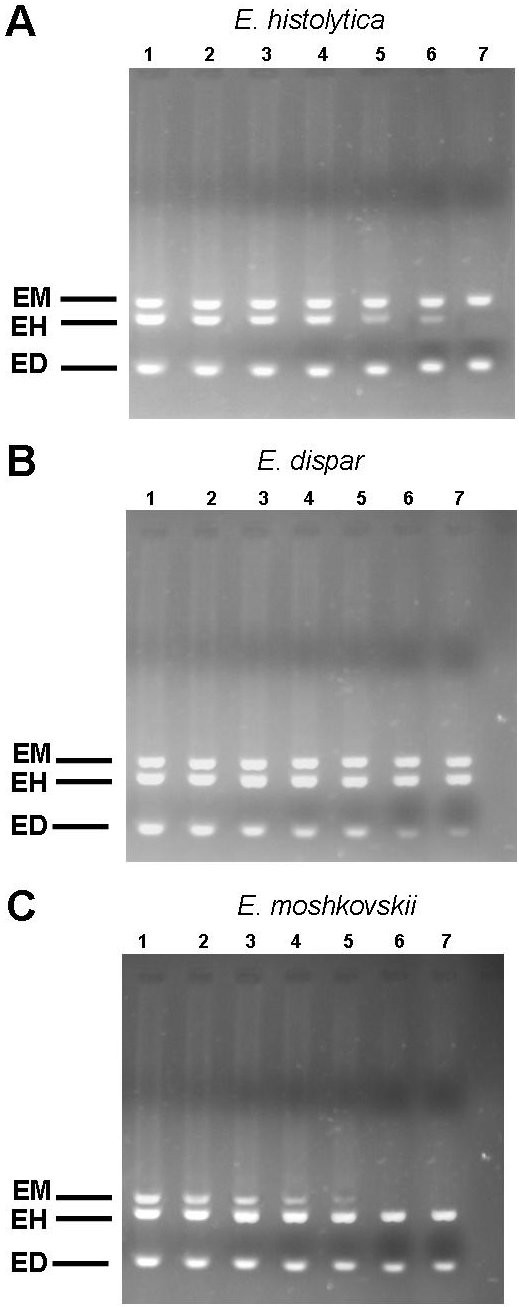
Detection of *E. histolytica *(A), *E. dispar *(B) and *E. moshkovskii *(C) in mixed cell lysates. To 1000 cells of *E. dispar *and *E. moshkovskii *(A) or *E. histolytica *and *E. moshkovskii *or (B), *E. histolytica *and *E. dispar *(C), 1000 cells (lane 1), 100 cells (lane 2), 10 cell (lane 3), 1 cell (lane 4), 0.1 cell (lane 5), 0.01 cell (lane 6) and 0.001 cell (lane 7) of the other species were added. Amplification was done by nested multiplex PCR.

1000 cells of *E. dispar *and *E. moshkovskii *species as the background allowed for the detection of a 0.01 cell of *E. histolytica*; 1000 cells of *E. histolytica *and *E. moshkovskii *species as the background allowed for the detection of a 0.001 cell of *E. dispar *; and 1000 cells of *E. histolytica *and *E. dispar *species as the background allowed for the detection of a 0.1 cell of *E. moshkovskii *(Figure 2).

#### Cross checking the results of nested multiplex PCR

The cross checking of the results of nested multiplex PCR was satisfactory as the same results were reproduced in randomly selected samples showing a mixed infection when subjected to individual species specific PCR in separate tubes.

#### Nested multiplex PCR

**Figure 3:**
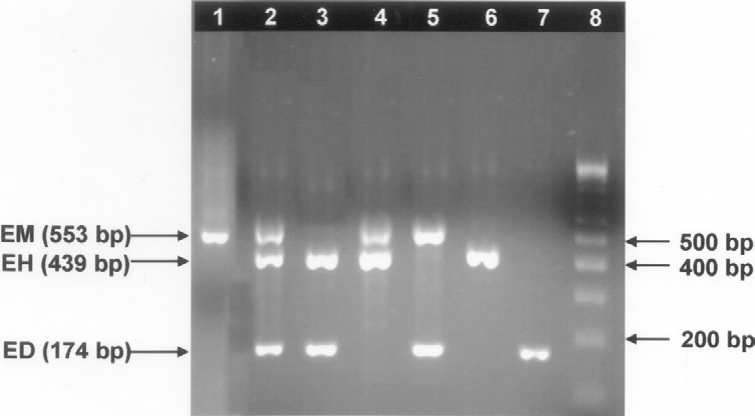
Differential detection of *E. histolytica*, *E. moshkovskii *and *E. dispar *by nested multiplex PCR on stool samples. The *E. moshkovskii *(EM), *E. histolytica *(EH) and *E. dispar *(ED) bands are 553, 439 and 174 bp, respectively. Lane-1, *E. moshkovskii *(mono infection); Lane-2, *E. moshkovskii*, *E. histolytica *and *E. dispar *(mixed infection); Lane-3, *E. histolytica *and *E. dispar *(mixed infection); Lane-4 *E. moshkovskii *and *E. histolytica *(mixed infection); Lane-5, *E. moshkovskii *and *E. dispar *(mixed infection); Lane-6 *E. histolytica *(mono infection), Lane-7 *E. dispar *(mono infection); Lane-8, 100 bp DNA ladder (Bangalore genei, Bangalore).

The nested multiplex PCR developed and evaluated in the present study showed that the size of diagnostic fragments of PCR products was clearly different for all the three *Entamoeba *species, the species-specific product size for *E. histolytica*, *E. moshkovskii *and *E. dispar *was 439, 553 and 174 bp respectively (Figure 3).

The nested multiplex PCR was performed on a total of 237 stool specimens including 202 stool specimens positive for *E. histolytica*, *E. dispar*, or *E. moshkovskii *by microscopy and/or culture, and 35 amoebae-negative control stool specimens. The nested multiplex PCR was positive in 190 out of 202 stool specimens that were positive for *E. histolytica*/*E. dispar/E. moshkovskii *complex trophozoites/cysts by microscopy and/or culture, thus showing a sensitivity of 94%. All the 35 negative control stool samples were negative by nested multiplex PCR thus showing a specificity of 100%.

The probability of negative nested multiplex PCR results in 35 control stool samples due to PCR inhibitors was ruled out by spiking the DNA of negative stool specimens with standard DNA of *Entamoeba *followed by nested multiplex PCR amplification.

The nested multiplex PCR detected mono-infection with *E. histolytica *in 7.4% (15 of 202), *E. dispar *in 49.5% (100 of 202) and *E. moshkovskii *in 1.0% (2 of 202) of stool samples. The PCR also detected mixed infections by both *E. dispar *and *E. moshkovskii *in 8.9% (18 of 202), *E. dispar *and *E. histolytica *in 18.8% (38 of 202), and *E. histolytica *and *E. moshkovskii *in 1.0% (2 of 202) of stool samples. The test also detected mixed infections by all the three species (*E. histolytica*, *E. dispar *and *E. moshkovskii*) in 7.4% (15 of 202) of stool samples (Table 1). The result of the nested multiplex PCR as compared with microscopy and culture is also summarized in Table 1.

PCR positivity of *E. dispar*, *E. histolytica *and *E. moshkovskii *among the stool samples that were positive for *E. histolytica/E. dispar*/*E. moshkovskii *by microscopy and/or culture is presented in Table 2.

### TechLab *E. histolytica *II ELISA

The TechLab *E. histolytica *II ELISA test was performed to detect *E. histolytica *coproantigen in 45 randomly selected stool samples that were positive for *E. histolytica*/*E. dispar*/*E. moshkovskii *complex by microscopy and/or culture.

The TechLab *E. histolytica *II ELISA detected coproantigen in 29 out of 45 (64.4%) stool specimens positive for *E. histolytica*/*E. dispar*/*E. moshkovskii *complex by microscopy and/or culture. These 29 amoebic antigen positive stool specimens included 4 specimens positive for *E. histolytica *as mono-infection, 17 specimens positive for *E. histolytica *and *E. dispar *as mixed-infection, 1 specimen positive for *E. histolytica *and *E. moshkovskii *as mixed-infection, and 7 specimens positive for all the three species namely *E. histolytica*, *E. moshkovskii *and *E. dispar *as mixed-infection by the nested multiplex PCR (Table 3).

Comparison of results of nested multiplex PCR and TechLab *E. histolytica *II ELISA test performed on 45 stool specimens is summarised in the table 3.

## Discussion

To be able to detect and distinguish *E. histolytica, E. dispar *and *E. moshkovskii *in stool samples is extremely important for accurate diagnosis of intestinal amoebiasis and for knowing the true prevalence of pathogenic *E. histolytica *in the community. Various DNA based molecular methods have been evaluated for accurate detection of *E. histolytica, E. dispar *or *E. moshkovskii *[2,6,9-16].

The present study, describes a new nested multiplex PCR strategy for species-specific detection and differentiation of *E. histolytica*, *E. dispar *and *E. moshkovskii *DNA directly in the stool samples of patients.

Recently a single round PCR to detect *E. histolytica*, *E. dispar *and *E. moshkovskii *in stool samples has been reported by Hamzah et al [17], the study reported that out of 27 stool samples positive for *Entamoeba *spp. by microscopy only 7 were successfully identified at species level by PCR, which included 1 positive for *E. histolytica *and 6 for *E. dispar*, but no amplification of *E. moshkovskii *was observed in a Thai population [17]. In contrast, our study has shown the presence of *E. moshkovskii *in the Indian population. The negative result for *E. moshkovskii *in Thai population may be attributed to the small sample size used in the study. A detailed comparative study between these two newly described PCR techniques may yield useful information especially in the field of molecular-based diagnosis of intestinal amoebiasis.

Nested PCR was used in the present study because it increases sensitivity [18]. Clinical specimens such as stool often contain PCR inhibitors even after purification steps. The two rounds of PCR might have assisted in compensating the effects of inhibitors present in clinical specimens. The first PCR product may be in too low concentration for detection with ethidium bromide stained gels using a UV transilluminator. The detection limit of agarose gel electrophoresis with ethidium bromide stain using an UV transilluminator is approximately 10 ng of DNA [19]. The product of first PCR may be just enough to provide adequate templates for the synthesis of second PCR product in the nested reaction to be detected by ethidium bromide staining.

The nested multiplex PCR was negative in 12 out of 202 stool specimens that were positive for *E. histolytica*/*E. dispar */*E. moshkovskii *complex trophozoites/cysts by microscopy and/or culture. The negative result due to inhibition of PCR in all these 12 stool samples was ruled out by spiking with standard DNA of *Entamoeba *followed by nested multiplex PCR amplification.

The negative PCR result in these 12 stool samples may be due to the presence of other *Entamoeba *species. However, we feel that this supposition needs to be proven by further development of molecular tools to confirm the presence of other *Entamoeba *species commonly found in humans, such as *E. coli*, *E. hartmanni *or other similar looking *Entamoeba *species. Till then these negative results may be imputed to the sensitivity limitation of the nested multiplex PCR technique.

In the present study by using nested multiplex PCR it was shown that the rate of mono infection with *E. dispar *was the highest. *E. dispar *was demonstrated in 100 out of 202 stool samples (49.5%) amongst patients attending JIPMER hospital. The study also shows that the rate of co-infection with *E. histolytica *and *E. dispar *was the highest (18.8%) as compared to both, *E. dispar *and *E. moshkovskii *(8.9%), and *E. histolytica *and *E. moshkovskii *(1.0%). The occurrence of co-infection with *E. dispar *and *E. histolytica *in the stool specimens has been documented by several studies reported earlier [9,10,12,20,21].

Nested multiplex PCR was positive for *E. histolytica *DNA in 6 stool specimens which were negative for coproantigen by TechLab ELISA. The possible reason for such occult infection (PCR-positive, ELISA-negative stool specimens for *E. histolytica*) may be due to degradation of lectin antigen in stool specimen due to prolonged storage (approximately 30–60 days) at -20°C prior to testing; thus resulting in a negative test for amoebic coproantigen by the ELISA.

In the present study the overall correlation between the results of nested multiplex PCR and that of TechLab *E. histolytica *II ELISA to detect *E. histolytica *in stool specimen was greater than 90%. This agreement between these two techniques shows clearly that either of the techniques may be used alone to yield an accurate assessment of the presence of *E. histolytica *in a stool specimen, but not for the detection of either *E. dispar *or *E. moshkovskii *in stool specimens by TechLab *E. histolytica *II ELISA.

The inability of TechLab *E. histolytica *II ELISA to detect either *E. dispar *or *E. moshkovskii *in stool specimens is the noted disadvantage of the test. The nested multiplex PCR, on the other hand appears to be more useful for simultaneous detection of all the three species,*E. histolytica*, *E. moshkovskii *and *E. dispar *when performed directly on the stool specimens. This is the main advantage of this test, and is of importance due to the fact that there is an increasing documentation of both *E. moshkovskii *and *E. dispar *from different parts of the world [2,6,9,11-17], including from Puducherry, the southern union territory of India [10]. The coexistence of non-pathogenic *E. dispar *and *E. moshkovskii *as mixed infection or solely as mono-infection amongst the patients showed an increased possibility of faulty diagnosis when the identification of *E. histolytica *was based primarily on morphology by microscopic examination of stool. The high PCR positivity of *E. moshkovskii *among the study population supports the view that humans are a true host for this free-living amoeba [2].

The inability to distinguish *E. histolytica *from those of morphologically similar *E. dispar *or *E. moshkovskii *in the stool samples is the main limitation of microscopy or culture. As shown in the present study only 34.6% of 202 stool specimens positive for *E. histolytica*, *E. dispar *or *E. moshkovskii *complextrophozoites/cysts by either microscopy or culture were actually *E. histolytica *and the remaining majority of stool specimens were positive for *E. dispar *and/or *E. moshkovskii*. In the absence of tests such as the nested multiplex PCR or ELISA for specific detection of *E. histolytica*, the majority of suspected infections would have been wrongly diagnosed as *E. histolytica *infection and treated unnecessarily with anti-amoebic drugs. We therefore, recommend the use of the nested multiplex PCR for simultaneous detection and accurate identification of all the three *Entamoeba *species in stool specimens.

## Conclusion

The present study reports a new nested multiplex PCR strategy for species specific detection and differentiation of *E. histolytica*, *E. dispar *and *E. moshkovskii *DNA in stool specimens. The test is highly specific, sensitive and also rapid; results of the test are available within 12 hours of receipt of stool specimens.

## Methods

### Sample details

A total of 1,755 stool samples were collected in the present study during a study period of two years from July 2004 to July 2006. This included 1,720 stool specimens collected from patients attending JIPMER hospital, Puducherry, India, with complaints of gastrointestinal discomfort. It also included 35 stool samples, as control samples, collected from healthy persons as well as patients with other intestinal infections. The 35 control stool samples included 15 stool samples negative for common enteric pathogens from healthy persons, 6 samples positive for enteric bacteria by bacterial culture, 10 positive for *Giardia intestinalis *cysts and 4 for eggs of *Ascaris lumbricoides*. All these 35 stool samples were negative for *E. histolytica*/*E. dispar*/*E. moshkovskii *complex cysts and trophozoites by microscopy and culture.

Fresh unpreserved stool samples were collected in sterile capped containers for examination by microscopy and culture for *E. histolytica*/*E. dispar*/*E. moshkovskii *complex. Aliquots of fresh unpreserved stool samples were stored at -20°C until used for PCR and ELISA tests.

### Microscopic examination of stool

Both saline and iodine wet mounts of fresh unpreserved stool samples were examined microscopically for demonstrating *E. histolytica*/*E. dispar*/*E. moshkovskii *complex cysts and trophozoites as previously described [22]. Briefly, saline wet mounts were made by mixing approximately 2 mg of stool with a drop of physiological saline on a glass microscope slide and placing a cover slip over the stool suspension. Similarly, iodine wet mounts were prepared by adding approximately 2 mg of stool to a drop of Lugol's iodine (diluted 1:5 with distilled water) on a glass microscope slide and placing a cover slip on the stool suspension. These wet mounts were microscopically examined initially by using a low-power (10×) objective and then using a high-power (40×) objective of a compound light microscope. The wet mount was read in approximately 5 min to view at least 100 fields per slide. Each stool sample was screened by at least three well trained microscopists before reporting negative results in our laboratory.

### Stool culture

Stool samples were cultured for *Entamoeba *species in Locke-egg (LE) medium (NIH modification of Boeck and Drbohlav's medium) within 6 h of collection as previously described [23,24].

### Nested multiplex- polymerase chain reaction (PCR)

#### Extraction of genomic *Entamoeba *DNA

The DNA was isolated from stool specimens by a cetyltrimethylammonium bromide (CTAB) extraction method modified from previously described method for DNA isolation from in vitro cultures of *Entamoeba *[25]. Briefly, 50 mg of stool specimen, was dispersed in 250 μl of lysis buffer (0.25% sodium dodecyl sulfate in 0.1 M EDTA, pH 8.0), and 100 μg/ml of proteinase K was added. The lysate was incubated at 55°C for 2 hours. Then 75 μl of 3.5 M NaCl followed by 42 μl of 10% CTAB/0.7 M NaCl (heated to 55°C) was added. After the components were mixed, the sample was incubated at 65°C for 30 min. This was followed by extractions with equal volumes of chloroform and then phenol-chloroform-isoamyl alcohol, and the DNA was precipitated with ice cold ethanol. The dried DNA pellet was dissolved in sterile distilled water and passed over a DNA clean-up spin column (Bangalore Genei KT-62, Bangalore). The DNA was finally eluted from the spin column in 100 μl of Tris-EDTA (TE) buffer; 2.5 μl of this DNA solution was used in the PCR reaction.

#### Quantification of DNA in stool specimen

Quantification of DNA in spin column purified DNA extract from stool specimens was determined by UV absorbance using a Cintra 5 double beam spectrophotometer. DNA yields were calculated on the basis of UV absorbance × dilution. The purity of the nucleic acid in the samples was estimated by the ratio of readings at 260 nm and 280 nm (OD _260_/OD _280_). The quantification of DNA was done only for representative stool specimens to know the DNA yielding capacity of the CTAB-DNA extraction method and also to estimate the purity of extracted DNA for its suitability to be used in PCR.

#### Primer design

The genus specific primers were designed using nucleotide sequences of 16S- like rRNA gene of *E. dispar*, *E. histolytica *and *E. moshkovskii *Laredo deposited in GenBank with accession number [GenBank:Z49256], [GenBank:X56991] and [GenBank:AF149906] respectively. The comparison of all the three 16S-like rRNA gene sequences of *E. dispar*, *E. histolytica *and *E. moshkovskii *Laredo revealed significant differences enough to design species specific primers. The primers were designed using Primer 3 on-line software [26]. The primer sequences used for nested multiplex PCR are shown in table 4.

#### Primer validation

The primer sequences designed for *E. moshkovskii*, *E. histolytica*, and *E. dispar *were subjected to a Basic Local Alignment Search Tool (BLAST) in the genome database of all organisms [27] and were found to be specific for the study.

The amplified PCR products of all the three species in stool samples were confirmed by getting both the strands of DNA sequenced on ABI PRISM 377 sequencer (Indian Institute of Science, Bangalore, India). Briefly, the ABI PRISM 377 DNA sequencer automatically analyzes DNA molecules labeled with multiple fluorescent dyes. After samples are loaded onto the system's vertical gel, they undergo electrophoresis, laser detection, and computer analysis. Electrophoretic separations are viewed on-screen in real-time. Software enables this system to support sequencing and fragment analysis applications. ABI PRISM 377 DNA sequencer can generate readings up to 900 bases per sample with 98.5% accuracy. The sequencing was done using species specific primers of each species. For example, the PCR product of *E. histolytica *DNA was sequenced using the species specific primer EH-1/EH-2, *E. dispar *using ED-1/ED-2 and *E. moshkovskii *using Mos-1/Mos-2.

The PCR product from stool samples showing mixed infection by multiplex PCR were sent for sequencing which included stool samples with mixed infection containing all the three species (*E. histolytica *+ *E. dispar *+ *E. moshkovskii*). Each species DNA was amplified separately with respective species specific primers before sending for sequencing. All sequences were analyzed for homology by using the nucleotide-nucleotide "BLAST" search feature [27]. The identities between the sequencing result of PCR product of *E. dispar*, *E. histolytica *and *E. moshkovskii *with the sequence deposited in GenBank, with accession number [GenBank:Z49256], [GenBank:X56991] and [GenBank:AF149906] respectively, were analyzed by using the "Align two sequences (bl2seq)" feature [27].

#### Standard strains

Three standard strains used in this study were *E. histolytica *HM-1: IMSS, *E. dispar *SAW760, and *E. moshkovskii *Laredo. These were used as a positive control in the present study. The lyophilized DNA of these strains was generously gifted by Dr. C. Graham Clark from London School of Hygiene & Tropical Medicine, London, UK.

#### Nested multiplex PCR protocol

For a reaction volume of 25 μl, comprising 2.5 μl of 10X PCR buffer (Biogene), 1.5 μl of 25 mM MgCl_2 _(Bangalore genei), 1.4 μl of deoxynucleoside triphosphate mix (5 mM each dNTP, ABgene), 0.3 μl (5 IU/μl) of *Taq *polymerase (Biogene), 0.3 μM of each primer (IDT) and 2.5 μl of template DNA was added in genus specific and species specific PCR. The PCR tubes were finally placed in an Eppendorf Thermal cycler [Master cycler gradient].

#### The conditions for genus specific PCR were as follows

The PCR mix was subjected to an initial denaturation at 96°C for 2 minutes, followed by 30 cycles – each consisting of 92°C for 60 seconds (Denaturation), 56°C for 60 seconds (Annealing), and 72°C for 90 seconds (Extension). Finally one cycle of extension at 72°C for seven minutes was performed. In the species specific nested multiplex PCR (which had multiple primer sets in the same tube), only the annealing temperature was changed to 48°C, leaving the other parameters of the amplification cycles unchanged.

Three micro litres of the amplification products were separated by electrophoresis through 1.8% agarose gel (Agarose Low EEO, Bangalore genie products, Bangalore, India) in 0.5 × Tris-borate-EDTA at 120 V for 45 min and were visualized by ethidium bromide staining under UV light for bands of DNA of appropriate sizes (Figure 3). Positive and negative control reactions were included with each batch of samples analyzed by nested multiplex PCR.

#### Assessment of competition for non-target DNA

To assess the competition of other non-target DNA present in stool samples with target DNA, the nested multiplex PCR was checked with reference DNA (DNA from standard strain of *E. histolytica*, *E. dispar *and *E. moshkovskii*) spiked with DNA from negative control stool samples (negative by microscopy and culture) followed by nested multiplex PCR amplification.

#### Estimation of minimum number of *Entamoeba *cells detectable by nested multiplex PCR

To determine the minimum number of *Entamoeba *cells detectable by nested multiplex PCR, all the three species were studied by Locke-egg (LE) medium (NIH modification of Boeck and Drbohlav's medium) cultures and the amoebae were counted using a standard haemocytometer. A cell pellet containing 10^6 ^cells was preferred for determining the detection limit of nested multiplex PCR for each *Entamoeba *species. The cell pellet containing 10^6 ^cells of *E. histolytica*, *E. dispar *and *E. moshkovskii *was diluted ten folds in Phosphate buffer saline (PBS) to obtain different concentrations of cells, such as 10^5^, 10^4^, 10^3^, 10^2 ^and 10 cells. The different quantities of cells ranging from 10^6 ^to 10 cells were added to 0.05 gm of faeces (negative control stool samples) followed by DNA extraction and PCR as per the aforementioned protocol.

#### Estimation of nested multiplex PCR to detect mixed infections with *E. histolytica*, *E. dispar *and *E. moshkovskii *species

A variable number of lysed *E. histolytica *cells (ranging from 1000 cells to 0.001 cell) were mixed with a constant number (that is 1000 cells) of each of the lysed *E. dispar *and *E. moshkovskii *cells; a variable number of lysed *E. dispar *cells (ranging from 1000 cells to 0.001 cell) were mixed with a constant number (that is 1000 cells) of each of the lysed *E. histolytica *and *E. moshkovskii *cells; a variable number of lysed *E. moshkovskii *cells (ranging from 1000 cells to 0.001 cell) were mixed with a constant number (that is 1000 cells) of each of the lysed *E. histolytica *and *E. dispar *cells, followed by DNA extraction and PCR as per the aforementioned protocol.

#### Cross checking the results of nested multiplex PCR

Some of the representative stool samples showing mixed infection by nested multiplex PCR were selected randomly and subjected to species specific individual nested PCR, with species specific primers for each species, in separate PCR tubes.

### TechLab *E. histolytica *II ELISA test

The TechLab *E. histolytica *II ELISA test was performed to detect *E. histolytica *coproantigen in 45 randomly selected stool samples, positive for *E. histolytica*/*E. dispar*/*E. moshkovskii *complex, by microscopy and/or culture, and also positive for *E. histolytica, E. dispar *and *E. moshkovskii *DNA by nested multiplex PCR. The TechLab *E. histolytica *II ELISA test was performed as per the instructions of the manufacturer. The kit was generously given for the purpose of research by TechLab, Inc. (Blacksburg, Va.).

## Authors' contributions

KK carried out the experimental works, and drafted the manuscript. SCP supervised and coordinated the study, and helped in the completion of the manuscript.

## Figures and Tables

**Table 1:** Comparison of results of nested multiplex PCR (Polymerase Chain Reaction), microscopy and culture on stool specimens.

Nested multiplex PCR result	Microscopy and culture result ^*a*^				Total no. of specimen
		
	Positive by microscopy and culture	Positive by microscopy only	Positive by culture only	Negative by microscopy and culture	
*E. dispar *(mono infection)	**80**	**16**	**4**	**0**	**100**
*E. histolytica *(mono infection)	**12**	**2**	**1**	**0**	**15**
*E. moshkovskii *(mono infection)	**2**	**0**	**0**	**0**	**2**
*E. dispar *+ *E. moshkovskii *(mixed)	**16**	**0**	**2**	**0**	**18**
*E. dispar *+ *E. histolytica *(mixed)	**35**	**2**	**1**	**0**	**38**
*E. histolytica *+ *E.moshkovskii *(mixed)	**2**	**0**	**0**	**0**	**2**
*E.dispar+E.histolytica+E.moshkovskii *(mixed)	**15**	**0**	**0**	**0**	**15**
Negative	**2**	**2**	**8**	**35**	**47**

Total	**164**	**22**	**16**	**35**	**237**

^*a *^Microscopy and culture was unable to distinguish between cyst and trophozoite of *E. histolytica*, *E. dispar *and *E. moshkovskii *in stool specimens.

**Table 2:** PCR positivity (%) of *E. dispar*, *E. moshkovskii *among the stool samples that were positive for *E. histolytica/E. dispar*/*E. moshkovskii *by microscopy and/or culture^*a*^.

Type of *Entamoeba *species	No. of samples positive by nested multiplex PCR	(%)
*E. moshkovskii*	37	2.1
*E. dispar*	171	9.9
*E. histolytica*	70	4.0

^*a *^Total number of stool samples collected from patients attending JIPMER hospital, Puducherry, India, with complaints of gastrointestinal discomfort = 1,720

**Table 3:** Comparison of results of nested multiplex PCR (Polymerase Chain Reaction) and TechLab *E. histolytica *II ELISA test performed on stool specimens.

Nested multiplex PCR result	TechLab *E. histolytica *II stool specimen test result^*a*^		Total no. of specimen
		
	Positive	Negative	
*E. dispar *(mono infection)	**0**	**8**	**8**
*E. histolytica *(mono infection)	**4**	**2**	**6**
*E. moshkovskii *(mono infection)	**0**	**0**	**0**
*E. dispar + E. moshkovskii *(mixed)	**0**	**2**	**2**
*E. dispar + E. histolytica *(mixed)	**17**	**1**	**18**
*E. histolytica *+ *E. moshkovskii *(mixed)	**1**	**0**	**1**
*E. dispar+E. histolytica+E. moshkovskii *(mixed)	**7**	**3**	**10**
Negative	**0**	**0**	**0**

Total	**29**	**16**	**45**

^*a *^*E. histolytica *II ELISA test is designed to detect only *E. histolytica *specific lectin antigen in stool specimen and therefore can confirm only the presence of *E. histolytica*.

**Table 4:** Primer sequences used for nested multiplex PCR

Genus specific primers (First PCR)	
*Entamoeba *genus	E-1 5' TAAGATGCACGAGAGCGAAA 3' (forward primer) E-2 5' GTACAAAGGGCAGGGACGTA 3' (reverse primer)

Species specific primers (Second nested multiplex PCR)	

*E. histolytica *species	EH-1 5' AAGCATTGTTTCTAGATCTGAG 3' (forward primer) EH-2 5' AAGAGGTCTAACCGAAATTAG 3' (reverse primer)
*E. moshkovskii *species	Mos-1 5' GAAACCAAGAGTTTCACAAC 3' (forward primer) Mos-2 5' CAATATAAGGCTTGGATGAT 3' (reverse primer)
*E. dispar *species	ED-1 5' TCTAATTTCGATTAGAACTCT 3' (forward primer) ED-2 5' TCCCTACCTATTAGACATAGC 3' (reverse primer)
